# Exploring the Functional Roles of Endophytic Bacteria in Plant Stress Tolerance for Sustainable Agriculture: Diversity, Mechanisms, Applications, and Challenges

**DOI:** 10.3390/plants15020206

**Published:** 2026-01-09

**Authors:** Akhila Sen, Johns Saji, Parammal Faseela, Chunquan Zhang, Shibin Mohanan, Ye Xia

**Affiliations:** 1 Department of Botany, Mar Athanasius College, Kothamangalam, Ernakulam 686666, Kerala, India; akhilasen@macollege.in; 2 Krieger School of Arts and Science, Johns Hopkins University, Baltimore, MD 21218, USA; johnsthevarpadam@gmail.com; 3 Department of Botany, Korambayil Ahamed Haji Memorial Unity Women’s College, Manjeri, Malappuram 676122, Kerala, India; faseela8888@gmail.com; 4 College of Agriculture and Applied Sciences, Alcorn State University, 1000 ASU Drive, Lorman, MS 39096, USA; czhang@alcorn.edu; 5 Department of Botany, Nirmala College, Muvattupuzha, Ernakulam 686661, Kerala, India; 6 Plant Pathology Department, The Ohio State University, 2021 Coffey Road, Columbus, OH 43210, USA

**Keywords:** endophytic bacteria, plant-microbe interactions, plant stress tolerance, sustainable agriculture, stress tolerance mechanisms, diverse stress conditions

## Abstract

Endophytic bacteria, which reside within plant tissues without causing harm, play crucial roles in promoting plant health and enhancing tolerance to biotic and abiotic stresses, making them highly valuable for sustainable agriculture. This review explores the diversity, mechanisms, applications, and challenges associated with endophytic bacteria in enhancing stress tolerance in plants. Endophytic bacteria display extensive diversity, spanning multiple phyla such as Actinobacteria, Bacteroidetes, Firmicutes, and Proteobacteria, each contributing uniquely to plant growth and stress tolerance. The functional mechanisms by which endophytic bacteria promote stress tolerance against biotic and abiotic stresses include the induction of plant systemic resistance, synthesis of bioactive compounds, competition for space and resources, nutrient production and transfer, etc. Despite their great potentials, challenges such as the complexity of plant–microbe interactions, variability in bacterial efficacy across different environmental conditions, and the need for advanced identification and application techniques hinder the widespread application of endophytic bacteria in agriculture. This review underscores the importance of harnessing the great potential of endophytic bacteria for developing sustainable agricultural practices and highlights the urgent need for further research to overcome existing challenges.

## 1. Introduction

Plant bacterial endophytes are plant-associated bacteria that inhabit internal tissues such as the stems, leaves, roots, seeds, flowers, and fruits, without causing harm or disease. They can be isolated from these tissues and are capable of colonizing the plant apoplast, intercellular spaces, and xylem vessels. Bacterial endophytes can play key roles in modulating host physiology rather than simply forming a generic “mutualistic” association [1]. Successful colonization is affected by various factors like host plant genotype, plant tissue type, bacterial taxon, strain type, and the other biotic and abiotic environmental conditions. Bacterial endophytes can generate extracellular enzymes such as cellulases, proteases, pectinases, and amylases that aid in their colonization of host plants. Bacterial endophytes can alter plant immune responses, allow successful colonization while avoid strong host defense activation, and thereby establish stable, compatible interactions with their hosts. They influence diverse host processes including immune signaling, growth and development, and resilience to both biotic and abiotic stresses [2,3]. According to Madbouly [4], endophytic bacteria can promote plant growth and yield, enhance pathogen suppression, support bioremediation, stimulate the synthesis of bioactive metabolites and induced systemic resistance (ISR). They can also improve the uptake of macro- and micro- nutrients in complex environments, for example, by solubilizing mineral forms or altering rhizosphere chemistry, which further supports plant nutrition. Many bacterial endophytes synthesize defensive secondary metabolites that protect plants against invading pathogens. For example, bacterial endophytes isolated from the roots and leaves of *Miscanthus giganteus* increased host resistance to the fungal pathogens through various metabolites production and/or activation of the host defense pathways [5]. Similarly, the bacterial endophytic strain *Bacillus velezensis LS123N* has been reported to effectively suppress rice blast, bacterial blight, bakanae disease, pythium disease, and brown spot, demonstrating the practical value of endophytes in disease management [6]. Bacterial endophytes can also enhance plant tolerance to abiotic stresses such as heavy metals, drought, salinity, extreme temperatures, and nutrient deficiencies. These bacterial endophytes enhance plant survival under adverse soil conditions by regulating plant water uptake and maintaining cellular water balance, modulating plant osmotic pressure, and scavenging stress-induced reactive oxygen species (ROS) driving by plants under diverse stress conditions [7,8].

In summary, plant associated endophytic bacteria are beneficial bacteria which can be applied in agriculture to enhance plant health and effectively manage both biotic and abiotic stresses. This review targets the diversity of endophytic bacteria, explores their underlying functional mechanisms of action, examines their agricultural applications, and discusses the challenges that must be addressed to fully harness their potential.

## 2. Endophytic Bacterial Diversity

Endophytic bacteria inhabit internal plant tissues, including roots, leaves, stems, flowers and seeds, where they form diverse communities composed of multiple bacterial phyla. This taxonomic diversity is both structural and functional, as each phylum contributes unique physiological and biochemical traits that collectively enhance plant growth, stress tolerance, and protection against pathogens and diseases. In particular, different phyla specialize in complementary mechanisms, such as biological nitrogen fixation, phytohormone production, osmoprotection, antioxidant activation, nutrient mobilization, and antagonism against pathogens. Consequently, the diversity of endophytic bacteria is closely associated with the range and effectiveness of plant stress-adaptive responses [9,10].

### 2.1. Proteobacteria

Proteobacteria is the most prevalent and functionally versatile phylum of endophytic bacteria. There are various classes in this phylum, and each has unique traits and functions:

- Alpha-proteobacteria: Rhizobium, Bradyrhizobium, and Agrobacterium are among the genera that belong to this class. Given their capacity to fix nitrogen and promote the growth of plants, Rhizobium species are essential for sustainable agriculture because of their symbiotic relationship with host plants, which not only promotes plant development by promoting the development of root nodules where they transform ambient nitrogen into ammonia—a type of nitrogen source that plants may easily use but also increases soil fertility [11].
- Beta-proteobacteria: This class comprises Herbaspirillum and Burkholderia, which are well known for plant growth-promoting and biocontrol properties. Burkholderia spp. colonizes plant roots and internal tissues, enhances nutrient acquisition, and produces lipopeptides and bioactive compounds that suppress pathogens, thereby reducing biotic stress. Herbaspirillum species are also known to fix nitrogen and stimulates phytohormone production to improve plant growth and stress tolerance [12].
- Gamma-proteobacteria: Pseudomonas and Enterobacter are two genera that belong to the class Gamma-proteobacteria. The ability of these bacteria to biocontrol plant diseases is well known. Many antimicrobial substances, such as hydrogen cyanide, phenazines, and pyoverdines, are produced by Pseudomonas species that can inhibit the growth of pathogenic microbes [13,14].

### 2.2. Actinobacteria

Another important class of endophytic bacteria is the actinobacteria, which are well-known for their ability to produce antibiotics and other bioactive metabolites [15]. Important genera in this phylum are Streptomyces and Micromonospora.

- Streptomyces: Streptomyces species generate a wide spectrum of antibacterial and antifungal compounds that suppress soil-borne pathogens and diseases and impart biotic stress tolerance. They play key role in decomposition and nitrogen cycling, improving soil organic matter quality and nutrient supply, which strengthens plant tolerance under various abiotic stresses [16].
- Micromonospora: Bioactive compounds produced by Micromonospora species protect plants against infections and promote plant growth. By breaking down complex organic molecules, they release nutrients that can be readily absorbed by plants. Additionally, these bacteria produce siderophores, which chelate iron and increase its availability to plants, enhancing their uptake of nutrients [17].

### 2.3. Firmicutes

This phylum comprises organisms known as core endophytes, which have the unique ability to thrive in various plant tissues and withstand harsh environmental conditions. Notably, genera such as Bacillus, Paenibacillus, and Staphylococcus have garnered significant attention for their capacity to promote plant growth and mitigate stress [18].

- Bacillus: The genus Bacillus comprises some of the most well-characterized endophytic bacteria, owing to their remarkable ability to form endospores. This attribute allows them to withstand extreme conditions, including drought, salinity, heat, and nutrient limitation. Bacillus spp. plays a crucial role in enhancing plant stress tolerance by producing phytohormones, osmoprotectants, and exopolysaccharides, which collectively promote root development and improve plant water retention in challenging environments [19].
- Paenibacillus: Paenibacillus spp. plays critical role in nitrogen fixation, phosphate solubilization, and extracellular enzyme production, which together alleviate the impacts of various abiotic stresses. Induced phytohormones and volatile organic compounds stimulate root system development and enhance tolerance to drought and salinity, while their consistent detection across plant tissues indicates a central role in structuring stress-adaptive endophytic consortia [20].
- Staphylococcus: Endophytic Staphylococcus spp. tolerates osmotic stress, desiccation and high salinity, and produce enzymes and metabolites that support nutrient cycling in stressed soils. Their documented antagonism against phytopathogens suggests an auxiliary role in plant defense, particularly under combined abiotic–biotic stress scenarios [21].

### 2.4. Bacteroidetes

The Bacteroidetes represent a significant category of endophytic bacteria. Genera like *Chryseobacterium* and plant growth is aided by *flavobacterium*, which participates in the nutrition cycle [22].

- *Chryseobacterium*: These endophytes secrete degradative enzymes that mineralize complex organic matter, improving nutrient availability in depleted soils. Many strains also synthesize antifungal compounds that protect plants from pathogenic fungi there by reducing disease-related stress [23].
- *Flavobacterium* species helps nutrient cycling through extracellular enzyme production and can promote root growth and water and nutrient uptake. Their activity improves soil structure and fertility, indirectly increasing plant tolerance to drought and nutrient stress [24].

### 2.5. Other Phyla

Although the other phyla, such as *Acidobacteria* and *Planctomycetes*, are less well known, they still contribute significantly to the overall functionality and diversity of the endophytic bacterial community of plants:

- *Acidobacteria*: Adapted to acidic environments, *Acidobacteria* contribute to decomposition of complex organic matter and nutrient release in acid soils, thereby improving plant nutrition under pH-stress conditions. Although their plant interactions are less understood, they are increasingly recognized as important for maintaining soil health and buffering plants against nutrient stress in acidic ecosystems [25].
- *Planctomycetes*: *Planctomycetes* possess unusual cell biology and versatile metabolism, enabling participation in nitrogen and carbon cycling within plant-associated habitats. Through biofilm formation and specialized pathways (e.g., lipid-related metabolism), they support root-associated nutrient transformations and contribute to long-term soil carbon turnover, indirectly enhancing plant tolerance to nutrient and environmental stresses [26].

### 2.6. Methods of Endophytic Bacteria Identification

The complex interactions between plants and endophytic bacteria have sparked widespread interest due to their potential benefits in agriculture, ecology, and biotechnology.

- Culture-Based Techniques: For the purpose of isolating and characterizing endophytic bacteria, culture-based techniques are indispensable. In order to prevent the contamination of endophytic bacteria and isolation of pure cultures require proper surface sterilization of plant tissues and then inoculation into specific culture media. Biochemical and physiological analysis can be used to define isolated strains, offering information on their metabolic capabilities and adaptabilities. Although culture-based methods provide insightful information, their limits in growing fastidious or uncultivable species may lead to an underestimation of bacterial diversity [27].
- Molecular and Genomic Techniques: High-throughput investigations of microbial communities have been made possible by advances in molecular biology and genomic techniques, which have significantly advanced the study of endophytic bacteria. The PCR-based methods such as 16S rRNA gene sequencing and ITS sequencing can be used to accurately identify and classify endophytic bacteria. While Fluorescence In Situ Hybridization (FISH) provides the identification and localization of specific bacterial taxa within plant tissues and Next-Generation Sequencing (NGS) and genomic technologies offer comprehensive insights into microbial diversity and community structure [28].
- Functional Analysis: Functional analytic techniques, such as transcriptomics, proteomics, and metabolomics approaches offer deeper insights into the dynamics and function of gene expression/function and metabolic activities of endophytic bacteria. For instance, proteome investigations clarify the functional protein activities and composition, and metabolic techniques reveal the metabolic ranges and potential function of endophytic bacteria. Plant–microbe interactions are governed by regulatory networks and patterns of gene expression that are revealed by transcriptomic research. These functional assessments give crucial information about the functional roles of endophytic bacteria in plant growth, ecosystem functioning, and stress responses [29].

## 3. Endophytic Bacteria Can Inhibit Pathogens Directly Through Different Mechanisms

**Figure 1:**
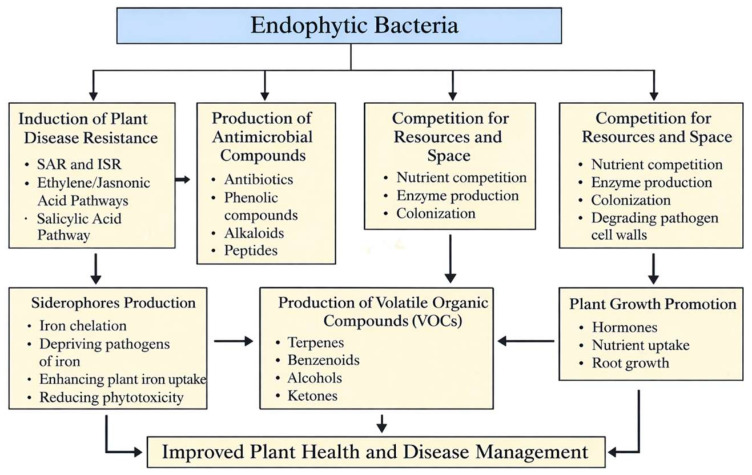
Endophytic bacteria improve plant health and growth by activating plant immune pathways, producing antimicrobial compounds and siderophores, competing with pathogens for resources, releasing growth-promoting and defensive VOCs, and enhancing nutrient uptake and hormone-mediated development.

Endophytic bacteria have evolved a mutually beneficial relationship with host plants throughout the course of long-term coevolution. Endophytic bacteria rely on plants for nutrients and living space, while plants benefit from endophytes to maintain their health. Numerous studies have conclusively demonstrated that endophytes enhance plant growth, strengthen host plant disease resistance, and improve tolerance to various abiotic stresses, such as drought, high temperatures and salinity, thereby promoting overall plant health (Figure 1). Many mechanisms have been discovered to prevent pathogens and diseases, including plant–pathogen competition for resources and niches, release of bioactive anti-microbial metabolites, induction of plant resistance, and promotion of plant development [30]. For instance, endophytic bacteria create a variety of metabolites that can both directly stop pathogen growth and help the host plants grow and thrive. Metabolites produced by endophytic bacteria can be applied to control plant pathogens and diseases, and their application to the induction of plant resistance shows great promises. In addition to producing secondary metabolites, such as growth hormones, bacterial strains can enhance the availability of crucial nutrients to promote plant growth [31]. In this section, we have documented the key mechanisms of pathogen/disease inhibition and plant growth promotion by endophytic bacteria (Figure 1).

### 3.1. Activation of Plant Systemic Disease Resistance

Two crucial plant systemic defense mechanisms against pathogen invasions are induced systematic resistance (ISR) and systemic acquired resistance (SAR). ISR is typically activated by beneficial microbes and relies primarily on ethylene (Eth) and jasmonic acid (JA) signaling. SAR is generally triggered by pathogens or chemical inducers and depends largely on the salicylic acid (SA) signaling pathway. Together, ISR and SAR constitute the core systemic resistance strategies in plants. Both ISR and SAR are long-lasting responses that provide broad-spectrum protection against a wide range of plant pathogens. SA is mainly involved in SAR for the control of biotrophic and hemibiotrophic pathogens. JA is mainly involved in ISR for the control of necrotrophic pathogens. In plants, generally, when the SA pathway is upregulated, the JA pathway is downregulated. However, there can also be synergistic interactions between these two hormone pathways. SA Mediated defense responses require the transcriptional co-activator NON-EXPRESSOR of PR 1 GENES (NPR1). While NPR1 is also required for ISR through the SA independent pathway [32].

SAR predominantly depends on salicylic acid accumulation and SA responsive gene expression. There is also interplay with L-Lys-derived immune signal N-hydroxypipecolic acid (NHP). Recognition of pathogen triggers the conversion of chorismate to isochorismate in the chloroplast via the stress-inducible ICS isoform ISOCHORISMATE SYNTHASE1 (ICS1). The recognition of pathogens triggers the increased SA biosynthesis, which activates the translocation of NPR1 from the cytosol to the nucleus, allowing it to bind TGA transcription factors and converting them into active regulators that drive defense gene expression and SAR. At the same time, this NPR1–TGA module suppresses JA-dependent transcription, thereby prioritizing SA-mediated immunity. In Arabidopsis, the Gretchen Hagen 3 (GH3) family acyl acid amido synthetase avrPphB SUSCEPTIBLE3 (PBS3) catalyzes an ATP dependent conjugation reaction between isochorismate and L glutamate, producing isochorismoyl L glutamate (IC Glu) as a biosynthetic intermediate. IC Glu is subsequently converted to salicylic acid through the removal of N pyruvoyl L glutamate (Pyr Glu), a step that occurs either spontaneously or is facilitated by the acyltransferases ENHANCED PSEUDOMONAS and ENHANCED PSEUDOMONAS SUSCEPTIBILITY1. Because PBS3 functions in the cytosol, isochorismate synthesized in the chloroplast must be exported to the cytoplasm for SA production. This transport is mediated by the chloroplast envelope localized multidrug and toxin extrusion (MATE) transporter ENHANCED DISEASE SUSCEPTIBILITY5 (EDS5), which plays a key role in enabling cytosolic SA biosynthesis [33,34,35].

During ISR, compared with uninfected plants, endophytic bacteria-inoculated plants generally show enhanced immunity to subsequent pathogen attack. For example, the rice bacterial endophyte *Azospirillum* sp. B510, originally isolated from *Oryza sativa cv*. *Nipponbare*, induces systemic resistance in its host by stimulating ethylene-dependent defense signaling. This resistance was effective against bacterial blight and rice blast disease, as demonstrated in a study by Kusajima et al. [31]. *Bacillus velezensis*, *Bacillus megaterium*, and *Herbaspirillum huttiense*, three endophytic bacteria recently identified in strawberry plants, were shown to enhance ISR and reduce the severity of *Rhizoctonia solani*-induced root-rot disease in tomato plants. The treated plants showed lower oxidative stress and higher antioxidant enzyme activities, along with increased proteins, carbohydrates, and phenolic compounds [32].

Plants show various molecular and cellular defense reactions during ISR activated by beneficial endophytic bacteria. These mechanisms involve activating the ethylene–jasmonic acid signaling pathway, promoting callose deposition, increasing reactive oxygen species (ROS) accumulation, upregulating defense-related genes that encode pathogenesis-related (PR) proteins, stimulating phytoalexin production, and regulating polyamine uptake, etc. These defense responses enhance the plants’ ability to resist pathogen attacks, making them more resistant to pathogens and diseases [32]. According to Asghari et al. [36], endophytic bacteria can activate ISR in grapevines against *Agrobacterium tumefaciens*. Root treatment with endophytic strains isolated from wild grapevines significantly enhanced the plant’s ability to withstand pathogen infection, largely by activating cellular defense responses, including the expression of pathogenesis-related (PR) genes. Similarly, induced resistance to rice sheath blight caused by *Rhizoctonia solani* was demonstrated in plants inoculated with the endophytic bacterial strain REB01, where increases in defense-related enzyme activities (such as peroxidase and polyphenol oxidase) and reductions in stress-related biochemicals like malondialdehyde were observed in rice leaves [37]. For some other studies, *B*. *subtilis* 11VM produced auxins and iturin, while *B*. *subtilis* 26 D secreted the lipopeptides surfactin and cytokinin to enhance the plant resistance against pathogen infections. Rumyantsev et al. [38] investigated the combined effects of the endophytic strains *B*. *subtilis* 11VM and *B*. *subtilis* 26D to increase the resistance of bread spring wheat to the greenbug aphid *Schizaphis graminum*.

Functional Genomic studies, such as RNA-seq data, has demonstrated that endophytic bacteria can induce the transcriptome reprogramming of defense genes in host plants for enhanced disease resistance [39]. For instance, RNA-seq profiling demonstrated that treatment with the *Bacillus BS-Z15 mycosubtilin* homologue elicits systemic resistance in *Arabidopsis* by activating transcriptional networks in plant–pathogen interaction pathways, MAP kinase signaling, and phenylpropanoid biosynthesis, with significant up-regulation of defense marker genes such as PR1 and PDF1.2 [40]. Similarly, RNA-seq analyses showed that root inoculation with *B*. *cereus NJ01* significantly alters host leaf transcriptomes, particularly salicylic acid and abscisic acid pathway genes. The EDS1/PAD4-WRKY18 regulatory complex drives the defense gene expression (e.g., ICS1, ABA biosynthesis genes) required for enhanced bacterial resistance [41]. In banana, the endophytic bacterium *Bacillus subtilis* TR21 induces transcriptomic reprogramming that enhances resistance to *Fusarium oxysporum* f. sp. cubense and promotes root growth by upregulating jasmonate and brassinosteroid biosynthesis pathways [42]. In tomato, endophytic *B*. *cereus BCM2* confers strong protection against the root-knot nematode Meloidogyne incognita, where RNA-seq–guided analyses identify WRKY transcription factors such as WRKY40 and WRKY32 as key candidate regulators of *Bacillus*-mediated induced systemic resistance and defense gene activation [43].

Host genotypes have a profound influence on root and rhizosphere associated microbiome composition, assembly, and functional interactions that shape stress responses such as disease and drought resistance/tolerance. For instance, in wheat drought tolerant and drought sensitive cultivars harbor distinct microbial communities with varying metabolic potential to modify host stress responses. This highlights plant genotype specific recruitment of beneficial microbiome [44]. Recent genomic studies show that endophytic bacteria establish mutualistic or defensive associations with plants through tightly regulated host processes that vary with host genetic makeup. For instance, it was reported that the endophytic bacterium *Streptomyces hygroscopicus* OSiSH-2 requires host cell ferroptosis to establish mutualism and enhance disease resistance in rice [45].

### 3.2. Production of Antimicrobial Compounds

Many secondary metabolites with antifungal and antibacterial characteristics are produced by endophytic bacteria in significant quantities, which are essential for suppressing microbial pathogen infections. These metabolites include antibiotics, phenolic compounds, alkaloids, peptides, etc. Antibiotics produced by endophytic bacteria can directly target pathogens by disrupting essential cellular processes, such as protein synthesis or cell wall formation. According to the previous study [45], endophytic bacteria found in a variety of medicinal plants have strong antimicrobial activity against a variety of pathogenic bacteria, including *Escherichia coli*, *Shigella flexneri*, *Proteus mirabilis*, *Klebsiella pneumoniae*, *Bacillus cereus*, *Citrobacter freundii*, etc. To combat pathogenic *Staphylococcus aureus*, *Escherichia coli*, *Bacillus subtilis*, *Pseudomonas aeruginosa*, and *Candida albicans*, naturally occurring endophytic bacteria *Bacillus mojavensis* and *Bacillus amyloliquefaciens* were isolated from the subsurface and upper parts of *Ajuga turkestanica* [46]. The study showed that antimicrobial metabolites produced by these endophytic bacterial strains can directly inhibit the growth and development of pathogenic microorganisms and are highly effective even at low concentrations [47]. Gram-positive bacteria, endophytic *Bacillus* and *Streptomyces* species from diverse habitats are well known for producing a wide range of antimicrobial compounds. Antibiotic compounds produced by endophytic bacteria interfere with vital cellular functions of pathogens, including cell wall formation, protein synthesis and DNA replication, ultimately causing cell death. For instance, phenolics and alkaloids can damage microbial cell membranes, inactivate enzymes, trigger oxidative stress, and disrupt nucleic acid functions of pathogens in plants. Additionally, antimicrobial peptides compromise membrane integrity and reduce pathogen virulence, thereby enhancing overall antimicrobial efficacy of plants [7]. These antimicrobial compounds provide natural protection against a wide range of plant pathogens, reducing the need for chemical pesticides and antibiotics in agriculture [48].

Through the production of bioactive metabolites, bacterial endophytes contribute to plant defense against pathogens and also represent a promising source of novel therapeutic agents. Currently, the majority of natural compounds generated from endophytes are used as bioactive commodities, such as antidiabetic, anti-viral, anti-cancer, antibacterial, and antifungal properties due to their diverse functional roles. For instance, the majority of bacterial endophytes isolated from *Cordia dichotoma* exhibited activity against the pathogens *Bacillus subtilis* and *Klebsiella pneumoniae*. *Dibutyl phthalate*, *eicosane*, *tetrapentacontane*, *heneicosane*, and *hexadecane* were the main antibacterial chemicals obtained from endophytic *Bacillus thuringiensis* [49]. Endophytic Bacillus thuringiensis, isolated from a variety of gymnosperms and angiosperms, was found to possess notable antibacterial activity [50].

### 3.3. Competition for Resources and Space

Endophytic bacteria help prevent plant pathogens from colonizing and proliferating within plant tissues by competing with them for nutrients and available space. By colonizing different parts of the plants, endophytic bacteria occupy ecological niches that would otherwise be available to pathogens. This competition deprives pathogens of essential nutrients and space required for their growth and proliferation. Bacterial endophytes are able to colonize a variety of plant tissues either locally or systemically [51]. Additionally, some endophytic bacteria possess specialized mechanisms, such as the ability to utilize specific nutrients more efficiently or produce enzymes that degrade pathogen cell walls, giving them a competitive advantage over pathogens [3]. According to Badran et al. [52], endophytic bacterial strains improved the nutritional efficiency of quinoa plants following *Pseudomonas syringae* infection, a pathogen that causes leaf spot disease, thereby supporting the continued growth of the seedlings.

The term “endophytic colonization” describes how endophyte populations enter, develop, and multiply within their host plants. For instance, the perennial plants *Boeravia erecta* and *Abutilon fruticosum*, respectively, are the source of endophytic strains of *Bacillus megaterium* and *Enterobacter hormaechei*, which colonize *Phaseolus vulgaris*, according to Mutungi et al. [53]. *Fusarium solani*, the pathogen that causes bean root rot, was lessened in severity and plant growth was promoted by the applications of these two strains. Additionally, endophytes generate a range of enzymes that help the plant cell wall break down gradually, which enables them to colonize plant surfaces. There are several different kinds of enzymes, including cellulases, chitinases, 1, 3-glucanases, and hemicellulases. These enzymes aid in the degradation of pathogen cell walls, which reduce the amount of phytopathogens in plant tissues. Furthermore, endophytic bacteria can modulate the host plant’s physiology to outcompete pathogens for resources. Due to their production of several secondary metabolites including phytohormones, endophytic bacteria are essential for promoting plant growth. The host plant’s general health and vigor can also be enhanced by certain endophytic bacteria through the stimulation of root development or/and improved nutrient uptake. Endophytic bacteria stimulate plant growth, thereby indirectly inhibiting pathogen growth and colonization [54].

### 3.4. Siderophores Production

Endophytic bacteria also produce siderophores, which increase plant development by binding to accessible iron, outcompeting phytopathogens for this element, and shielding the host plants from pathogen infection. With molecular weights ranging from 500 to 1000, siderophores are relatively small organic chelators that form Fe^3+^ siderophore complexes when they bind to insoluble iron in the environment. Siderophore-producing endophytic bacteria enhance the availability of iron in plant rhizospheres and deliver the essential iron for plant growth by chelating siderophores, which also modifies the quantity of iron in the soil [55]. In addition to being effective virulence factors, siderophores are produced and excreted by helpful bacteria, which serves as a mechanism for depriving harmful species of iron. Plant growth and development are enhanced by bacterial siderophores in the same way that they promote iron uptake [56]. Some recognized endophyte-produced siderophores, such as hydroxymate, phenolate, and/or catecholate types, have the ability to impart biocontrol activities. They can chelate iron from the environment and have a strong affinity for Fe^3+^, which gives bacteria access to iron. Typically, siderophores interact with iron to form complexes, which, either directly or through ligand exchange, contribute to iron uptake. Moreover, siderophores help iron-deficient plants to fix nitrogen since diazotrophic organisms require Fe^2+^ and Mo components for the production and functioning of nitrogenase [57]. Apart from this, siderophores can also form stable complexes with radioactive U or heavy metals like Cu, Zn, Cd, or Pb. When heavy metal pollution occurs, this procedure increases plant growth and decreases phytotoxicity [58].

According to Singh and Dubey [59], several endophytic actinomycetes, such as Streptomyces sp. mhcr0816, Streptomyces sp. UKCW/B, Streptomyces sp. GMKU 3100, and Nocardia sp., can produce siderophores. Furthermore, Streptomyces acidiscabies E13 is a particularly efficient siderophore producer that enhances the growth and development of *Vigna unguiculata* under nickel stress [60]. A siderophore from the marine endophyte *Pseudomonas aeruginosa* Mgrv7 has recently been shown to have anticandidal action [61]. The endophytic bacterial strain *Bacillus halotolerans* Q2H2 in potato plants exhibits a growth-promoting mechanism against fungal pathogens, as revealed by Wang et al. [62]. This mechanism involves the production of hydroxamate-type siderophores, encoded by the genes fhuC, fhuG, fhuB, and fhuD.

### 3.5. Production of Volatile Organic Compounds

Endophytic bacterial strains are reported to release volatile organic compounds (VOCs), which show great potential for promoting plant growth and inhibiting pathogen growth and colonization [63,64]. Microbial VOCs are small molecules that belong to several chemical classes, including terpenes, benzenoids, alcohols, ketones, organic acids, sulfur compounds, and pyrazines [29]. These VOCs can interfere with pathogen signaling pathways, disrupt cellular processes, or directly inhibit pathogen growth. *Pseudomonas*, *Bacillus*, *Enterobacter*, *Arthrobacter*, and *Burkholderia* species all can generate VOCs, including 2,3-butenediol and acetoin. In plant species, these VOCs alter the expression of genes related to development and defense, such as genes relevant to pathogenesis, diverse plant metabolic pathways, emergence of systemic resistance, and absorption of nutrients [65].

*Pseudomonas* species, which are the most well-studied endophytic bacterial strains, release volatile organic compounds (VOCs) that can cause host plants to become resistant to a range of bacterial and fungal pathogens. Examples of these VOCs include acetophenone, 1,2-benzisothiazol-3(2H)-one, 2-undecanone, 1,3-butadiene, dimethyl disulfide, benzaldehyde, dimethyl trisulfide, nonanal, N,N-dimethyldodecylamin, isovaleric acid, benzothiazole, cyclohexanol, 2-ethyl 1-hexanol, 3,5,5-trimethyl-1-hexanol, n-decanal, decyl alcohol, etc. [66]. *Ralstonia solanacearum R32*, the cause of potato bacterial wilt disease, was suppressed in its growth by a variety of volatile organic compounds (VOCs) produced by endophytic *Bacillus* strains, including B. *aerius Kh867*, *B*. *pumilus Fer469*, *B*. *zhangzhouensis Kh690*, *B*. *safensis Har267*, and B. *wiedmannii Ah945* [67]. Moreover, Ghazala et al. [68] reported that VOCs released by *Bacillus mojavensis I4* inhibited the development of phytopathogens and meanwhile promoted the growth of *Arabidopsis thaliana*. VOCs can boost plant development, inhibit pathogens and diseases, and function as signaling molecules in the interactions between plants and microorganisms. Several bacterial VOCs have been shown to exhibit strong nematicidal, antibacterial, and pesticidal activities, as well as the ability to induce plant tolerance, enhance defense responses, and promote growth.

## 4. Endophytic Bacteria Induced Abiotic Stress Tolerance

**Figure 2:**
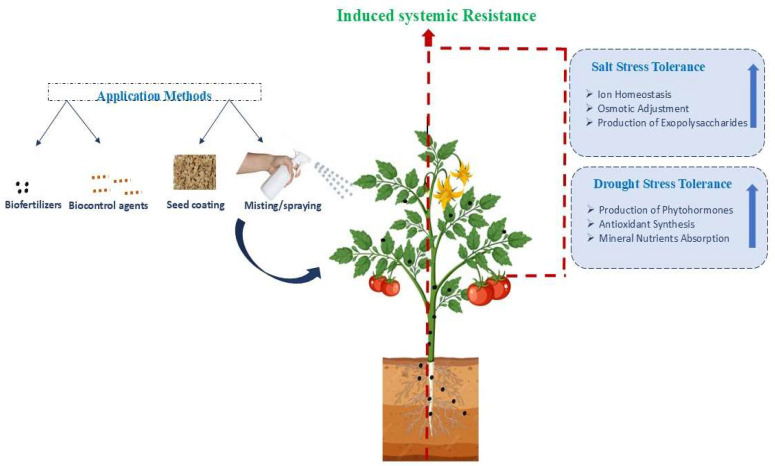
Applications and mechanisms of endophytic bacteria in inducing drought and salinity stress tolerance in host plants. Blue arrows indicate application methods, and red arrows show root-to-shoot systemic signaling leading to ISR and improved salt and drought stress tolerance.

Abiotic stressors and rapidly changing climate patterns pose significant challenges to achieving optimal agricultural productivity on a global scale. Among the various abiotic stresses, salinity and drought are the most prevalent abiotic stress constraints affecting plant growth and yield. Exposure to stress conditions often leads to excessive generation of ROS in plants which disrupt cellular homeostasis, impair physiological processes, and ultimately leads to cell death [69]. Endophytic bacteria can be applied through various methods, including biofertilizers, biocontrol formulations, seed coating, and foliar misting or spraying, enabling their colonization of plant roots and internal tissues (Figure 2). Once established within the plants, these beneficial microbes activate induced systemic resistance (ISR) and trigger multiple stress-alleviating pathways. Under salinity stress, endophytes can contribute to ion homeostasis, osmotic adjustment, and the production of exopolysaccharides. Under drought stress, they promote the synthesis of phytohormones, enhance antioxidant production, and improve mineral nutrient absorption. Together, these mechanisms strengthen plant resilience and maintain growth under adverse environmental conditions (Figure 2).

### 4.1. Role in Salt Tolerance

The functional roles of endophytic bacteria in enhancing plant tolerance to salinity help mitigate the negative effects of soil salinity [70]. Endophytic bacteria, such as *Acidovorax facilis*, *Bradyrhizobium*, and *Rhizobium*, actively contribute to ion homeostasis in plants by modulating the movement of ions across plant cell membranes [71]. This regulation helps maintain a balanced intracellular ion environment by limiting sodium ion accumulation and promoting potassium uptake. Furthermore, these bacteria act as sodium ion sinks, sequestering excess sodium within their own cells, thereby reducing the overall sodium concentration in plant tissues [72]. By influencing ion transporters and channels, these bacteria play crucial roles in enhancing plant salt tolerance, paving the way for sustainable agriculture in saline environment [73]. Several mechanisms enable endophytic bacteria, such as *Acidovorax facilis*, *Bradyrhizobium*, and *Rhizobium*, to enhance plant salt tolerance.

Ion Homeostasis: Ion imbalances and toxicity can occur in plant cells exposed to saline conditions. Excessive sodium accumulation specifically disrupts cellular processes and inhibits plant growth. However, endophytic bacteria like *Acidovorax facilis*, *Bradyrhizobium*, and *Rhizobium* can help plants maintain ion homeostasis in these environments [74,75]. These bacteria facilitate the removal of toxic sodium and promote the uptake of essential potassium by modulating the activity of ion transporters and channels in plant cell membranes. Furthermore, they can sequester surplus sodium within their own cells, thereby reducing overall sodium levels in plant tissues [76].

Osmotic Adjustment: salinity stress induced by high salt concentrations in the soil can lead to water loss from plant cells [77]. To mitigate this, certain endophytic bacteria, such as *Rhodococcus rhodochrous*, produce osmoprotectants like proline and trehalose [78]. These osmoprotectants help the host plants maintain cell turgor and prevent dehydration in saline conditions. Functioning as small organic molecules, osmoprotectants can accumulate within cells to significant levels without disrupting normal cellular functions. Furthermore, they can protect proteins and membranes from damage caused by high salt concentrations and scavenge reactive oxygen species generated under stress [79].

Production of Exopolysaccharides: In saline environments, endophytic bacteria produce exopolysaccharides, forming a hydrogel matrix in the soil. This matrix enhances the soil’s water retention capacity, improving water availability for plants, which is particularly beneficial in water-scarce dry and semi-arid regions [80].

### 4.2. Role in Drought Tolerance

Several bacterial genera, including *Pseudomonas*, *Enterobacter*, *Pantoea*, *Stenotrophomonas*, *Acinetobacter*, and *Serratia*, are key to mitigating the adverse impacts of water limitation on plant development [81]. These bacteria improve plant resilience to drought stress through mechanisms such as producing plant growth-promoting hormones like auxins and cytokinins, which stimulate root development and control water absorption. Furthermore, some endophytic bacteria produce exopolysaccharides, improving soil water retention and enhancing water availability to plants [10]. By reducing transpiration and increasing water uptake, these bacteria demonstrate great potential as valuable tools for addressing the challenges posed by water scarcity, ultimately supporting resilient and sustainable agricultural practices [82].

Production of phytohormones: under drought stress, endophytic bacteria can synthesize phytohormones that benefit plant survival. For example, *Pseudomonas putida* produces auxins, promoting lateral root and root hair growth, which expands the root surface area for the improved water absorption and utilization. Furthermore, these bacteria can influence stomatal closure, reducing water loss through transpiration [83,84].

Antioxidant activities: endophytic bacteria can trigger antioxidant production in host plants, mitigating oxidative stress caused by drought. Drought typically induces oxidative stress, leading to cellular damage and inhibited growth in plants. By stimulating antioxidant synthesis, endophytic bacteria can protect plant cells from this damage. For example, *Bacillus subtilis* and other endophytes can promote antioxidant production in the host plants. These bacterial-induced antioxidants counteract drought-related oxidative stress, safeguarding plant cells. For instance, *Bacillus subtilis* can enhance the activity of antioxidant enzymes like catalase and superoxide dismutase within the host plants [85].

Mineral Nutrition Absorption: drought stress often impairs a host plant’s capacity to absorb essential minerals; however, endophytic bacteria can play crucial roles in mitigating this limitation. These bacteria enhance the plants’ access to vital soil nutrients, thereby helping to sustain plant health and productivity under water-scarce conditions [86]. For instance, the endophytic bacterium *Burkholderia phytofirmans* promotes phosphorus uptake in its host. Phosphorus, a key element for plant growth and particularly important for energy transfer within plants, becomes more accessible due to the presence of *Burkholderia phytofirmans*, which contributes to the improved plant health and productivity during drought stress [56].

ACC Deaminase Activity: ethylene elevations can impede the growth of plants and are frequently linked to stress reactions. 1-aminocyclopropane-1-carboxylate (ACC) deaminase is produced by some endophytic bacteria, such as *Pseudomonas fluorescens*. ACC, a precursor for the synthesis of plant hormone ethylene, is broken down by this enzyme. Therefore, this enzyme lowers the amounts of ethylene in plants, which lessens the detrimental effects of drought stress [87]. An overview of different endophyte species, associated host plants, and their roles in promoting plant growth and stress resistance is provided in Table 1.

## 5. Endophytic Bacteria and Soil Health

Endophytic bacteria also play critical roles in maintaining soil fertility and the overall health of ecosystems. After successful colonization, bacterial endophytes stimulate the growth and physiology of host plants as well as the properties of soil through direct and indirect mechanisms. Several endophytic bacterial taxa have been shown to improve soil health in association with specific host plants. For instance, species of *Bacillus* isolated from *Solanum nigrum* promote the hyperaccumulation of heavy metals such as Cd, Cu, and Cr, thereby facilitating phytoremediation and mitigating metal toxicity [106]. Similarly, *Burkholderia cepacia* residing in *Zea mays* contributes to the degradation of organic pollutants, including toluene and phenols, in contaminated soils [107]. Members of the genera *Enterobacter*, *Microbacterium*, and *Pantoea* are frequently associated with improved uptake, translocation and phytostabilization of heavy metals-particularly Cr, while also supporting nutrient cycling in grass-dominated systems [108]. In addition, *Serratia* and *Arthrobacter* spp. Isolated from *Brassica juncea* enhance soil organic matter dynamics, aid in the detoxification of inorganic pollutants such as vanadium and collectively promote plant growth and soil health, thereby reinforcing the multifunctional role of bacterial endophytes in soil health management [109].

Although some endophytic bacteria possess the enzymatic machinery required for biological nitrogen fixation, they represent only a small fraction of the total endophytic community. In rice the strains of *Burkholderia*, *Herbaspirillum*, *Azospirillum*, and *Rhizobium leguminosarum bv*. *trifolii* can supply approximately 19–47% of plant nitrogen via N_2_ fixation in internal tissues [110]. However, the consortia comprising *Gluconacetobacter diazotrophicus*, *Herbaspirillum seropedicae*, *H*. *rubrisubalbicans*, *Azospirillum amazonense*, and *Burkholderia spp*. contribute around 29% of the nitrogen requirement in sugarcane [111]. Phosphorus solubilization and mineralization are key microbial processes that increases its availability. Endophytic and rhizosphere-associated bacteria can mediate these processes by acidifying the rhizosphere by secreting organic acids such as carboxylic acids, which release phosphorus from bound forms, particularly Ca-associated phosphate in calcareous soils. Studies on *Solanum nigrum* and *ginseng* have shown that a subset of isolated endophytic strains possess efficient phosphate-solubilizing activity, demonstrating that endophytic bacteria can significantly increase plant-available phosphorus and therefore improve crop productivity [112,113].

These studies indicate that endophytic bacteria are integral determinants of soil health, functioning as a key regulator of phytoremediation, nutrient cycling, and plant productivity in diverse agroecosystems. By mediating biological nitrogen fixation, mobilizing poorly available phosphorus, and driving the phytoremediation of organic and inorganic pollutants, these symbionts simultaneously sustain soil fertility and reduce reliance on synthetic agrochemicals. Consequently, strategic exploration and deployment of functionally efficient endophytic strains represent a promising avenue for developing resilient, low-input, and environmentally sustainable cropping systems.

## 6. Applications of Endophytic Bacteria in Agriculture

**Figure 3:**
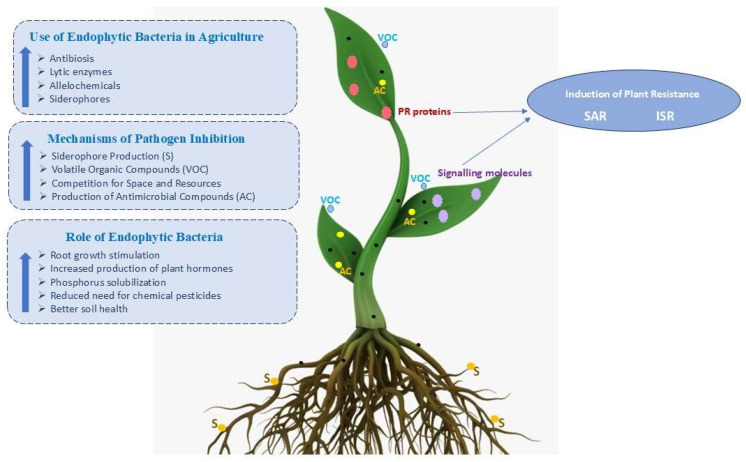
Role and application of endophytic bacteria in agriculture. Arrows represent systemic signalling from the site of induction to other plant parts, resulting in SAR and ISR.

Endophytic bacteria promote plant growth by synthesizing phytohormones such as auxin and gibberellin, enhancing nutrient uptake, and improving the bioavailability of nutrients. They also increase plant resistance to biotic and abiotic stresses, such as pathogens, pests, drought, and salinity, through inducing systemic resistance (ISR) and producing stress-alleviating compounds, for example, [114]. Additionally, endophytic bacteria can support sustainable agriculture by reducing the dependence on chemical fertilizers and pesticides, thereby reducing the harmful impact on environments and maintaining plant and soil health. Their ability to colonize internal plant tissues leads to long lasting interactions that strengthen plant resilience and productivity. Advances in biotechnology, formulation, and delivery methods have further enabled their applications as biocontrol agents, biostimulants, and biofertilizers, offering effective and eco-friendly approaches for enhancing plant and soil health and developing sustainable agriculture. Formulating these beneficial endophytic bacteria for agricultural applications is a rapidly growing research area with a great potential for promoting sustainable agricultural practices [86]. The effectiveness of their establishment within plant hosts is greatly influenced by the delivery methods. Common formulations include liquid suspensions, granules, wettable powders, and encapsulated forms (e.g., alginate beads), which improve bacterial stability and shelf life. These formulations are applied using different methods, such as seed coating, foliar spraying, soil drenching, or in planta inoculation during early plant development stage [105]. The role and applications of endophytic bacteria in agriculture are summarized in Figure 3.

Vertical transmission, where bacteria are transferred from parent plants to offspring via seeds, is a key delivery mechanism. This provides a head start by ensuring the presence of beneficial microbes from germination onwards [9]. For instance, the endophytic bacterium *Herbaspirillum seropedicae*, found in sorghum, rice, and maize seeds, promotes plant growth by producing plant hormones and facilitating nutrient uptake [115]. Horizontal transmission, another mode of acquisition, allows each plant generation to acquire endophytic bacteria from their surrounding environment, introducing potentially advantageous novel bacterial strains. For instance, *Burkholderia phytofirmans*, an endophytic bacterium colonizing grapevines through horizontal transmission, enhances plant health and productivity by increasing resilience to environmental stress [116]. Each formulation and delivery system presents distinct advantages and disadvantages. While vertical transmission ensures early colonization with beneficial bacteria, it may limit bacterial diversity. Horizontal transmission allows for the introduction of new strains but relies on their availability in the surrounding environment. Root colonization is a popular method, but it involves competition with other rhizosphere microorganisms. Inoculation through wounds or natural openings allows for targeted delivery but may induce stress in the plant [117].

One prevalent method for introducing endophytic bacteria in agriculture is seed coating. This process involves applying a bacterial formulation to seeds before planting. This ensures that the bacteria begin supporting the plants from initial stages of germination. For instance, maize seeds coated with the endophytic bacterium *Bacillus subtilis* have exhibited improved germination rates and enhanced seedling vigor. This early interaction initiates a symbiotic relationship that can improve the plant’s growth and disease resistance throughout its life cycle [118]. Another delivery technique involves directly spraying the bacterial liquid formulation onto plants or soil. This method is particularly effective for disease and pest management. For instance, research on grapevines demonstrated that spraying a formulation of the endophytic bacterium *Bacillus amyloliquefaciens* can significantly reduce downy mildew, a common grapevine disease [119]. By delivering the bacteria directly to the area of need, this method optimizes their distribution and effectiveness.

Root drenching is a further common delivery method to apply liquid bacterial suspensions directly to the soil near the plant roots. Plants release root exudates, such as organic acids, proteins, and amino acids, which act as signals attracting bacterial endophytes. These signals guide bacteria to the roots, where they colonize and establish a symbiotic relationship with the plants. The endophytic bacterium *Pseudomonas putida* can colonize maize roots, boosting growth and disease resistance [120]. *Pseudomonas putida* strain KT2440 can enhance tomato and cucumber root development and protect these plants against Fusarium wilt by producing siderophores and antibiotics to suppress soilborne pathogens. Their liquid suspensions can be applied through root drenching in crops such as tomato and cucumber [121], where fluorescent pseudomonads have been widely used as effective biological control agents against soil-borne pathogens.

Granular formulation of endophytic bacteria to the rhizosphere is also applicable. For instance, *Burkholderia phytofirmans* strain PsJN is a well-studied endophytic bacterium, which can enhance plant growth and stress tolerance under adverse conditions, such as salinity, drought, and cold. It colonizes internal plant tissues, improving photosynthetic efficiency and root architecture. It can be formulated as granules, such as biochar-based or polymer-encapsulated forms. This formulation can provide a sustained release of the bacterium, protecting it from environmental stress and facilitating effective root zone colonization. Its granular applications to the rhizosphere of plants, such as maize, grapevine, and wheat, have demonstrated improved root development and greater resilience to biotic and abiotic stress [122,123].

Post-harvest treatment of endophytic bacteria can improve plants’ shelf life without harmful chemical applications. *Bacillus velezensis* is an endophytic bacterium widely recognized for its effective biocontrol activity against post-harvest fungal pathogens. This bacterium can produce antifungal lipopeptides, such as bacillomycin and iturin, which can disrupt pathogen cell membranes to kill the pathogens. It is commonly formulated as a liquid suspension or wettable powder for post-harvest applications. It can be sprayed or dipped directly onto fruits and vegetables to inhibit fungal pathogens, such as *Penicillium*, *Botrytis*, and *Fusarium*. For example, studies had shown that the treatment of citrus fruits with *B*. *velezensis* significantly reduced blue and green mold incidence during storage, effectively extending shelf life without the use of chemical fungicides [124].

## 7. Environmental Issues, Challenges, and Limitations

The introduction of endophytic bacteria to new environments can disturb the existing microbial ecosystems. This disturbance has the potential to result in the displacement of indigenous beneficial microorganisms, which might potentially diminish soil vitality and biodiversity. For example, the introduction of non-native strains can lead to the displacement of indigenous bacteria, which in turn can cause changes in soil nutrient cycles and plant–microbe interactions [9]. Horizontal gene transfer (HGT) is the process by which genes, particularly those that confer antibiotic resistance, are transferred to other microbes in the soil by endophytic bacteria. The process of gene flow presents substantial hazards to both the well-being of the environment and human health. Agricultural soils have been shown to contain antibiotic resistance genes, which has raised concerns about the potential long-term effects of utilizing genetically engineered or naturally occurring antibiotic-resistant endophytes [56]. Releasing endophytic bacteria into the environment may result in unanticipated ecological ramifications. For example, a genetic variation that improves the ability of one type of plants to withstand dry conditions may also make it more vulnerable to attacks from pests or diseases in another type of plants, so impacting the local ecosystems and agricultural yields [115].

Identifying suitable endophytic bacterial strains that are compatible with particular crops and the unique soil conditions in a given area is a difficult task. The efficacy of strains can significantly differ according to environmental conditions like soil pH, temperature, and moisture levels [116]. A bacterial strain that is advantageous for one plant species may not provide the same benefits for another; hence, the field use of endophytic bacteria requires thorough field studies. The regulatory framework governing the approval and utilization of endophytic bacteria in agriculture is intricate and differs across different regions. Strains are subjected to thorough testing to ascertain that they do not present any hazards to human health or the environment, a process that may be both time-consuming and expensive. Expanding the production of endophytic bacteria for commercial purposes poses considerable difficulties. Ensuring the survival and activities of bacteria throughout the process of formulation, storage, and transportation is of utmost importance. Furthermore, maintaining consistent active populations and performance in various agricultural environments necessitates the implementation of strong quality control systems [125].

Although there has been a significant amount of study conducted, there is still a lack of complete understanding regarding the detailed functional mechanisms by which endophytic bacteria enhance plant development and stress tolerance. This discrepancy impedes the advancement of more precise and efficient bacterial strains [9]. Endophytic bacteria may exhibit variable performance in field situations as opposed to controlled laboratory settings. The efficacy of bacterial inoculants can be influenced by the variability in soil qualities, climate conditions, and plant genotypes, which poses challenges in predicting outcomes [126]. The expenses associated with the development, testing, and commercialization of endophytic bacterial products can be too high, to the point of preventing or hindering their progress. Small-scale farmers, particularly those in developing regions, often face knowledge and financial constraints that limit their ability to access and adopt these biotechnological solutions, thereby hindering their widespread acceptance [127].

## 8. Conclusions and Future Prospectives

Endophytes, including endophytic bacteria, are currently seen as very promising sources for improving plant and soil health due to their vast diversity and effective functions. They contribute to sustainable agriculture by promoting plant growth as well as enhancing plant tolerance to abiotic and biotic stresses. Utilizing beneficial endophytic bacteria–plant interactions can significantly enhance the health and yield of both food and non-food crops, promoting sustainable agriculture with minimal resource input and reducing the harmful chemical applications. Although the stable, multi-generation inheritance of beneficial endophytic genome components remains a major challenge, targeted microbiome engineering and the rational assembly of endophyte consortia are emerging as powerful approaches to enhance crop performance and yield. Within broader agroecological and regenerative agriculture frameworks, endophytic bacteria can be positioned as core biological inputs that enhance ecosystem functions rather than merely as stand-alone bioinoculants. By promoting nutrient cycling, improving soil structure, suppressing pathogens and increasing crop resilience to climatic stresses, they complement practices such as diversified rotations, cover cropping, reduced tillage, organic amendments, etc. When selected and applied in alignment with these practices, preferably as consortia tailored to specific crops and environmental contexts, bacterial endophytes can help close nutrient loops, reduce reliance on synthetic fertilizers and pesticides, and accelerate soil regeneration. In doing so, they help translate key principles of agroecology and regenerative agriculture into practical, plant-integrated and microbe-driven strategies.

Optimizing the beneficial potential of plant–endophytic bacteria interactions requires a thorough understanding of the functional mechanisms that enable endophytic bacteria to interact with plants and play their beneficial functions. Even though they are widely distributed and can be found in great quantities, they frequently do not yield reliable results in the field. One reason for this discrepancy could be our poor understanding of the complex dynamics underpinning plant–endophytic bacteria relationships under different environmental conditions. Successful formation of plant–endophytic bacteria relationships is largely dependent on plant genotypes and environmental conditions. By establishing functional plant–endophyte relationships under controlled conditions before field introduction, more consistent field trials can be achieved. Numerous higher plants flourish in various unconventional habitats, occupying distinct ecological niches. Therefore, techniques like micropropagation and vegetative propagation, which generate genetically identical host plants, can be used to minimize the effects of host genetic variation on plant–endophytic bacteria interactions. Additionally, there is a high likelihood that bacteria from unexplored wild plants will be unique and interesting, as the diversity of endophytes has not been extensively researched. The unique endophytes in wild plants may help them endure harsh environments and combat biotic and abiotic stresses. To identify these rare and promising bacterial endophytes that have wide-ranging positive effects on plants, a mix of methods that rely on both culturing and non-culturing techniques would be necessary. At the same time, several unresolved controversies that limit the potential use of endophytic bacteria in the field. For instance, some endophytic bacterial strains can shift along the mutualist–pathogen continuum under climate or management stress, raising concerns about context-dependent pathogenicity. Strong defense induction may also impose resource-allocation trade-offs with growth such that net benefits depend on host nutrition, stress intensity and developmental stage. Furthermore, emerging evidence for heritable epigenetic effects on plant–microbe fidelity suggests that long-term stability and transmission of beneficial endophytes across generations may be less straightforward than currently assumed, and these issues warrant targeted investigation in future work [124,127].

Future research needs to be focused on unraveling the functional mechanisms and applications of plant–endophytic bacteria interactions under different environmental conditions. The plant associated endophytic bacterial microbiome enhances host functions, promoting plant growth and health under unfavorable conditions. Microbiome engineering holds a strong potential as a transformative strategy for developing sustainable crop production. The development of crop-specific endophytic bacterial consortia, including diverse endophytic bacteria with different modes of action, will be critical for enhancing the related beneficial effects. Also, Microbiome genes (M genes) serve as key targets for shaping plant-associated microbial communities. Understanding plant–microbiome interactions at the community level, supported by advanced omics tools and microbiome engineering, can provide valuable insights into microbiome assembly and its functional feedback on plant growth and defense [128]. Host-directed microbiome engineering is the critical area for systemic investigation. For instance, previous research demonstrate that plant associated microbiomes are actively shaped by host genotype, immune signaling, and metabolic programs rather than functioning as microbe driven assemblages, underscoring the need for host informed microbiome engineering strategies [129,130,131,132]. Future studies should integrate plant genetics, immune regulation, and environmental context to design host directed approaches that enable predictable recruitment, stability, and function of beneficial microbiomes in agricultural systems. In addition, the delivery systems of these formulated endophytic bacteria need to be optimized to maintain bacterial population and activities to improve their consistency and efficacy in practical applications. Also, a major gap in the related field is the lack of concrete milestones to bridge laboratory discoveries with field applications of beneficial microbial consortia. Future efforts should establish standardized in planta efficacy screening protocols and integrated multi omics validation pipelines to ensure strain functionality and reproducibility under realistic conditions. In parallel, clear regulatory roadmaps are needed to translate engineered microbial consortia into deployable and commercially viable agricultural solutions. These future studies will improve our understanding of how to harness the full potential of endophytic bacteria to enhance plant health and yield in sustainable farming systems, benefiting both the environment and a rapidly growing global population.

## Figures and Tables

**Table 1:** An overview of various endophytic species, their associated host plants, and their contributions to plant growth promotion and stress resistance.

Endophyte Species	Host Plant	Activity/Effects	References
*Microbacterium* sp.	*Arabis alpine*	Under significant metal stress, produced phytohormones and enhanced plant development.	[88]
*Sphingomonas*, *Steroidobacter* and *Actinoplanes*	*Nicotiana tabacum*	Enhanced Cd stress resistance.	[89]
*Enterobacter mori*, *E*. *ludwigii*, *E*. *cloacae*, *Bacillus amyloliquefaciens*, *B*. *siamensis*, *Pseudomonas rhodesiae* and *Kosakonia oryzae*	*Oryza officinalis*	Encouraged the development of robust root systems, biomass accumulation, chlorophyll content, and nitrogen uptake in plants.	[90]
*Bacillus halotolerans*	*Solanum tuberosum*	Antagonistic effect on pathogenic fungi and reduced the plant diseases and enhanced plant growth.	[91]
*Curtobacterium oceanosedimentum*, *Curtobacterium luteum*, *Enterobacter ludwigii*, *Bacillus cereus*, *Micrococcus yunnanensis* and *Enterobacter tabaci*	*Oryza sativa*	Produced a variety of phytohormones that reduced the negative effects of NaCl stress and improved rice development parameters.	[63]
*Bacillus safensis*	*Nerium indicum*	Drought protective effect.	[92]
*Microbacterium albopurpureum*	*Chiloschista parishii*	Plant growth promotion.	[93]
*Bacillus velezensis*, *Bacillus megaterium* and *Herpaspirillum huttiense*	*Fragaria chiloensis*	Serve as both a plant growth inducer and therapeutic nutrients to combat Rhizoctonia root rot disease.	[94]
*Bacillus subtilis*	*Triticum aestivum*	Reduced the toxic effects of cadmium stress.	[32]
*Stenotrophomonas rhizophila*	*Glycyrrhiza uralensis*	Reduced the disease index of Cucumber *Fusarium* wilt, promoted seed germination, enzyme activities and seedling growth.	[95]
*Rahnella Victoriana* and *Bacillus paramycoides*	*Brassica napus*	Enhanced the crop yields and alleviated arsenic accumulation.	[96]
*Bacillus subtilis*	*Triticum aestivum*	Resistance against greenbug aphid *Schizaphis graminum*.	[97]
*Sphingomonas paucimobilis*	*Dendrobium officinale*	Promoted plant growth and resistance to salt, drought and cadmium stress.	[38]
*Bacillus subtilis*	*Stipa purpurea*	Resistance towards high temperature, low temperature and ultraviolet stress.	[98]
*Burkholderia* sp.	*Nicotiana tabacam*	Resistance towards *Ralstonia solanacearum*, a bacterial pathogen causing bacterial wilt disease.	[99]
*Pseudomonas oryzihabitans*	*Pelargonium graveolens*	Significant rise in the secondary metabolite production and plant growth.	[100]
*Salinicola salarius*	*Avicennia officinalis*	By phosphate solubilization and the hormone (Indole 3-acetic acid) that promotes plant development, resistance to severe temperatures and salt tolerance is achieved.	[101]
*Paenibacillus peoriae*	*Triticum aestivum*	Enhanced plant growth and resistance towards phytopathogens such as *Fusarium culmorum*.	[102]
*Paenibacillus polymyxa*	*Endostemon obtusifolius*	Drought stress mitigation and plant growth enhancement effects.	[103]
*Bacillus mycoides*	*Viburnum grandiflorum*	Amelioration of heavy metals zinc and nickel stress.	[104]
*Bacillus safensis*	*Raphanus sativus*	Alleviated salt stress and increased plant yield.	[105]

## Data Availability

No new data were created or analyzed in this study.
